# Supplementary material for: Associations between lung function and physical and cognitive health in the Canadian Longitudinal Study on Aging (CLSA): A cross-sectional study from a multicenter national cohort
Source: PLoS Med. 2022 Feb 9;19(2):e1003909. doi: 10.1371/journal.pmed.1003909 (PMC8870596; doi:10.1371/journal.pmed.1003909)
Supplement: S1 Protocol — (DOCX) [file pmed.1003909.s001.docx]

**S1: Protocol of planned analysis submitted to Hamilton Integrated Research Ethics Board**

**Background and study relevance.**

Spirometry is an effort-dependent test, which measures lung capacity, an important indicator of lung health. This information is pivotal in the diagnosis of respiratory diseases. However, lung capacity alone has also been shown to be an important and independent predictor of all-cause mortality and cardiovascular events (CVD)[1-5]. Furthermore, there is emerging data linking lung function to the development of other chronic diseases such as strokes, diabetes mellitus, cancers and renal diseases [6,7]. This suggests that lung capacity may serve as a broad indicator of general health and survival and not just lung health. Such a measure can be useful in the risk stratification of premature death, CVD events and development of certain chronic diseases. Furthermore, the underlying mechanistic links for these associations are not well understood and have not been extensively examined.

We propose to utilise the CLSA spirometry data, which is the largest spirometry dataset collected to date from the Canadian population with sufficient representative numbers for the ages of 45 to 85 years, to examine the prevalence and phenotypes of impaired spirometric lung function in the general population and how it relates to symptoms, general health and functional status.

**Relevance:** These findings will provide a better understanding on the broader implications of spirometry and aging. It addresses a number of gaps in knowledge regarding the relationship between lung capacity (spirometry), morbidity, physical impairment and disability.

**Study Objectives/ Hypothesis:** We hypothesized that spirometry is a simple and sensitive measure of a highly complex integrative process involving the neuromuscular axis, muscle strength, chest-wall mechanics and lung mechanics. Each component needs to operate efficiently individually and together to provide the optimal conditions for oxygen extraction and delivery to meet the body’s metabolic demand. However, the aging process and other systemic pathological conditions can adversely impact on the structural integrity and function of any one or more of these individual components, leading to alterations in the overall measured ventilatory capacity parameters on spirometry. We postulate that in certain individuals, who experience accelerated aging, a process that causes excessive structural and physiological decline to one or more of these components, can lead to excessive decline in lung capacity than otherwise be expected for normal aging. At the same time, the accelerated aging process increase the risks for mortality and development of chronic diseases. Therefore, spirometry may serve as an important marker of accelerated aging, and allows the identification of individuals who are vulnerable to excessive morbidity and mortality. The Canadian Longitudinal Study on Aging (CLSA) cross-sectional baseline data can provide further insights into this proposed mechanism and the broader implications of impaired lung function with aging.

**The objectives for this proposal are:**

1. To establish age- and sex- appropriate spirometric reference values using the large CLSA population-based spirometric database that is representative in numbers for the age groups of 45- 85 years and the current demographics of the Canadian population.

2. To derive appropriate thresholds for spirometric impairment and severity that relates to co-morbidity, and physical impairment.

3. To examine the prevalence of spirometry impairment and the phenotypes (obstructive versus non-obstructive) and how the phenotype relates to morbidity.

**Study design, methodology, and data analysis proposed:** Data from the 30,000 participants with spirometry measurements will be included in this proposal. Listed below are the steps that will be undertaken for validation of data quality and statistical analysis. (1) Validation of spirometry quality: As recommended by international guidelines, the quality of the spirometry needs to be individually verified by inspecting the spirographs generated following each effort [9]. If available, these will be inspected to ensure they meet the quality criteria for acceptability and reproducibility by international guidelines. For the higher grade measurements (as assessed by the spirometer software, grades A-C), 30% will be randomly selected for manual inspection by a trained personnel in spirometry reading. An agreement rate between the assessments by manual inspection and the spirometer of >90% will be considered acceptable. For the lower grades (grades D-F), all measurements will be inspected to select out the acceptable values measured with each effort. If spirographs are not available then plausibility of the data will be determined based on the participants age, sex, height and ethnicity. In this case, only grades A-C measurements will be used. Based on the published literature, it is anticipated that up to 30% of spirometry performed outside pulmonary function laboratory setting are considered suboptimal and excluded [11]. Therefore, we anticipate that the final sample size to be approximately 20,000 participants. Those failing to reach international guidelines standards will be explored to see if the same consequences apply i.e. are the standards excessively stringent. This issue is important to address, since the higher standards/guidelines currently being followed may be a major reason why spirometry is not being widely adopted and implemented in the community. Perhaps less stringent quality criteria may be more appropriate if the outcome for its intended use i.e. assessment of morbidity and mortality is the same. (2) Establishing age-, sex-appropriate reference values: A non-linear multiplicative regression model, which adjusts for height, age, sex and ethnicity that we have derived and validated in another large population-based study, PURE [12], will be used to generate reference equations for the CLSA population. These equations will provide the predicted/expected values for the FEV1, FVC and FEV1/FVC according to the height, age, sex and ethnic distribution in the CLSA cohort. The measured FEV1, FVC and FEV1/FVC for each participant will be compared to their predicted values and expressed as a percentage of the predicted (i.e. %pred = measured/predicted x 100). These %pred values provide an indication of the impairment level relative to the overall adjusted population average. A Z-score will then be calculated for each %pred value. The characteristics of participants with a Z-score of 0 or higher (i.e. population mean and higher) will be examined to determine the appropriate selection criteria that enables the selection of participants with the highest adjusted spirometric values in the population. The %pred values generated with this method will then be compared to the traditional selection criteria (i.e. based on self-reported never-smoking and no comorbidities). The relationship between the %pred derived from each method (population-average reference vs healthy cohort-reference), to symptoms, physical impairment and healthcare utilization will be examine to provide face validity to each method. (3) Establishing clinically-meaningful thresholds for diagnosing respiratory impairment that relates to symptoms, physical impairment and health care utilization. Hierarchical logistic regression will be used to establish the adjusted odds ratios (ORs) for symptoms (COPD and heart questionnaires), exercise tolerance (physical activity, 4 minute walk test), functional impairment (IAL, ADL, functional status), health resource utilization (receiving formal and informal care, hospitalization, medication use), across the Z score categories of the ‘population-average reference’. This will provide the magnitude of the relative risks of morbidity associated with each level of lung function impairment (%predFEV1, %predFVC) below the population average; and the relationship across the ORs across different severity of lung function impairment (i.e. linear trend in ORs or other pattern). By examining the relationship between the ORs for morbidity by lung function level will help determine the appropriate threshold to define impairment (4) Defining the different spirometric phenotypes and their relationship to co-morbidities. Spirometric impairment phenotypes will be examined using the threshold established from objective 3 above together with the FEV1/FVC ratio threshold for obstructive (i.e. FEV1/FVC <0.7) vs non-obstructive (>=0.70) phenotypes. Hierarchical logistic regression will be used to examine for any relationship between spirometric phenotypes and self-reported diagnoses of cardiovascular, strokes, neurological, auto-immune/ connective tissue diseases, osteoporosis, osteoarthritis, falls, medication use, hospital utilization. This exploratory analysis will provide insight into the relationship between multi-morbidity and clustering of certain co-morbidities to spirometric impairment phenotypes and the possible underlying mechanistic links/pathways.

**Sample size justification:** With the population-average reference method all high quality acceptable spirometry will be included, which is anticipated to be 20,000 participants. For the healthy-cohort reference methods, it is anticipated that 30% will be excluded due to smoking, symptoms or self-reported comorbidities giving an estimated sample size of 16,000. This sample is at least 10 fold larger than the present Canadian reference values [10] and therefore should provide enough power and robustness to our findings.

**References**

1. Beaty TH, Cohen BH, Newill CA, Menkes HA, Diamond EL, Chen CJ. Impaired pulmonary function as a risk factor for mortality. Am J Epidemiol.1982; 116: 102-113.

2. Beaty TH, Newhill CA, Cohen BH, Tockman MS, Bryant SH, Spurgeon HA. Effects of pulmonary function on mortality. J Chronic Dis.1985;38:703-710.

3. Friedman GD, Klatsky AL, Siegelaub AB. Lung function and outcome of myocardial infarction. N Engl J Med.1976; 295:1323.

4. Hole DJ, Watt GC, Davey-Smith G, Hart CL, Gillis CR, Hawthorne VM. Impaired lung function and mortality risk in men and women: findings from the Renfrew and Paisley prospective population study. BMJ.1996;313:711-715.

5. Kannel WB, Lew EA, Hubert HB, Castelli WP. The value of measuring vital capacity for prognostic purposes. Trans Assoc Life Insur Med Dir Am.1980;64:66-83.

6. Tockman MS, Anthonisen NR, Wright EC, Donithan MG. Airways obstruction and the risk for lung cancer. Ann Intern Med.1987;106:512-518.

7. Sin DD, Anthonisen NR, Soriano JB, Agusti AG. Mortality in COPD: role of comorbidities. Eur Respir J.2006;28:1245-1257.

8. Hutchinson J. On the capacity of the lungs, and on the respiratory functions, with a view of establishing a precise and easy method of detecting disease by the spirometer. Medico-Chirurgical Transactions (London).1846;29:137-161.

9. Miller MR, Hankinson J, Brusasco V, Burgos F, Casaburi R, Coates A, et al. Standardization of spirometry. Eur Respir J.2005;26:319-338.

10. Tan WC, Bourbeau J, Hernandez P, Chapman K, Cowie R, FitzGerald MJ, et al. Canadian prediction equations of spirometric lung function for Caucasian adults 20-90 years of age: Results from the Canadian Obstructive Lung Disease (COLD) study and the Lung Health Canadian Environment (LHCE) study. Can Respir J.2011;18:321-326.

11. Coates AL, Graham BL, McFadden RG, McParland C, Moosa D, Provencher S, et al. Spirometry in primary care. Can Respir J.2013;20:13-22.

12. Duong M, Islam S, Rangarajan S, Teo KK, O’Byrne PM, Schunemann HJ, et al. Global differences in lung function by region (PURE): an international, community-based prospective study. Lancet Respir Med.2013;1:599-609.

**Amendments to the protocol:**

December 2017: Some of the planned outcome variables such as healthcare utilization, medication use and activities of daily living were not available at the time and therefore were omitted from the analyses.

October 2019: Following the recommendations from ATS, which endorsed the use of the Global Lung Function Initiative (GLI) predictive values for the interpretation of FEV1 and FVC, we elected to remove objective 1 which was to establish our own predictive values using the CLSA spirometry data. Instead we used the GLI predictive values to interpret and grade the severity of FEV_1_.

January 2020: we decided to report on the graded relationship between FEV1 categories with outcomes rather than establishing thresholds since the relationship we observed was continuous throughout the range of the FEV1.

November 2021: Unadjusted analyses were conducted as requested by reviewers from PLOS Medicine and analyses on the FVC
