# Supplementary material for: Associations between lung function and physical and cognitive health in the Canadian Longitudinal Study on Aging (CLSA): A cross-sectional study from a multicenter national cohort
Source: PLoS Med. 2022 Feb 9;19(2):e1003909. doi: 10.1371/journal.pmed.1003909 (PMC8870596; doi:10.1371/journal.pmed.1003909)
Supplement: S1 Table — (DOCX) [file pmed.1003909.s003.docx]

**S1 TABLE.** Baseline Characteristics of the comprehensive and tracking cohorts.

|  | **Comprehensive cohort** | **Tracking cohort** |
| --- | --- | --- |
| N, % | 22,822, (100%) | 21,241 (100%) |
| Female | 11,981 (52%) | 10,835 (52%) |
| Age, yrs 45-54 | 6,235 (44%) | 5,832 (36%) |
| 55-64 | 7,769 (30%) | 6,564 (31%) |
| 65-74 | 5,396 (16%) | 4,634 (20%) |
| 75+ | 3,422 (10%) | 4,211 (13%) |
| Height, m (sd) | 1.69 (sd 0.1) | 1.70 (sd 0.3) |
| BMI, kgm^-2^ <25 | 7,082 (33%) | 7,758 (38%) |
| 25-30 | 9,226 (40%) | 8,232 (38%) |
| >30 | 6,496 (26%) | 5,142 (24%) |
| Education Primary | 1,074 (4%) | 1,986 (8%) |
| Secondary/Trade | 2,114 (9%) | 2,882 (14%) |
| University | 19,602 (87%) | 16,290 (78%) |
| Smoking Never | 7,318 (34%) | 6,239 (31%) |
| Former | 13,515 (57%) | 12,630 (58%) |
| Current | 1,851 (8%) | 2,267 (11%) |
| Physical activity | 153.2 (sd 76.6) | 151.2 (sd 78.8) |
| COPD | 1,040 (4%) | 1,436 (5.7%) |
| Asthma | 2,940 (13%) | 2,347 (11.3%) |
| CVD | 2,593 (9%) | 2,925 (11.7%) |
| No chronic conditions | 3,297 (20%) | 3,214 (18%) |
| >=3 chronic conditions | 8,664 (35%) | 8,736 (38%) |

CLSA recruited a stratified random sample of 51,338 participants aged 45 to 85 years old at baseline from 11 centers across 7 provinces of Canada. Among these, a subset of 30,097 (comprehensive cohort) individuals residing within 50km of each center were invited to attend in-person for comprehensive testing to gather physical, performance and clinical data. The remaining 21,241 participants (tracking cohort) were followed via phone visits to gather questionnaire data. The above data are from participants with no missing information are provided as frequencies (% of total column) for categorical variables or means (sd) for continuous variables. BMI= body mass index calculated as weight (kg) divided by height (m) squared; physical activity score was calculated using the PASE instrument with higher scores indicating higher physical activity; asthma, COPD (chronic obstructive pulmonary disease), CVD (cardiovascular disease) and chronic conditions were all obtained from self-reported interview-based questionnaires at baseline.
