# Supplementary material for: Associations between lung function and physical and cognitive health in the Canadian Longitudinal Study on Aging (CLSA): A cross-sectional study from a multicenter national cohort
Source: PLoS Med. 2022 Feb 9;19(2):e1003909. doi: 10.1371/journal.pmed.1003909 (PMC8870596; doi:10.1371/journal.pmed.1003909)
Supplement: S2 Table — (DOCX) [file pmed.1003909.s004.docx]

**S2 Table.** Contraindications to performing spirometry

| Severe acute respiratory condition |
| --- |
| Pregnancy (over 27 weeks) |
| Unstable heart disease |
| Heart surgery within the last 3 months |
| Major surgery on chest or abdomen within the last 3 months |
| Detached retina or recent eye surgery in the last 3 months |
| Blood in sputum in last 3 months |
| Thoracic, abdominal or cerebral aneurysm(s) present |
| Average blood pressure of 200/120 or above |
