# Supplementary material for: Associations between lung function and physical and cognitive health in the Canadian Longitudinal Study on Aging (CLSA): A cross-sectional study from a multicenter national cohort
Source: PLoS Med. 2022 Feb 9;19(2):e1003909. doi: 10.1371/journal.pmed.1003909 (PMC8870596; doi:10.1371/journal.pmed.1003909)
Supplement: S3 Table — AO, airflow obstruction; FEV1, forced expiratory volume in 1 second; FVC, forced vital capacity; LLN, lower limit of normal. (DOCX) [file pmed.1003909.s005.docx]

**S3 Table.** Adjusted analyses on self-reported respiratory symptoms, self-perceived poor health status, cognitive and physical performance for different grades of low FVC compared to reference group (FVC>0sd) in the overall cohort and in participants without spirometry airflow obstruction (shown here as FEV_1_/FVC >=LLN).

|  | **Categories of FVC according to GLI z-scores** | | | |
| --- | --- | --- | --- | --- |
|  | **>0sd** | **0 to >-1sd** | **-1 to >-2sd** | **=<-2sd** |
| Total 22,822 | 8,754 | Mild 9,387 | Moderate 3,884 | Severe 797 |
| **Perceived poor health** | | | | |
| 1,736 (7.6%) | 439 (5%) | 672 (7%) | 455 (12%) | 170 (21%) |
| OR (95%CI) | 1 | 1.19 (1.01, 1.41) p=0.036 | 1.53 (1.27, 1.85) p<0.001 | 2.22 (1.66, 2.96) p<0.001 |
| FEV_1_/FVC >=LLN | 1 | 1.19 (1.00, 1.41) p=0.046 | 1.54 (1.27, 1.86) p<0.001 | 2.38 (1.74, 3.24) p<0.001 |
| **Moderate-Severe symptoms** | | | | |
| 5,375 (24%) | 1,660 (19%) | 2,210 (24%) | 1,158 (29%) | 347 (44%) |
| OR (95%CI) | 1 | 1.18 (1.07, 1.30) p=0.001 | 1.41 (1.25, 1.60) p<0.001 | 2.51 (1.99, 3.17) p<0.001 |
| FEV_1_/FVC >=LLN | 1 | 1.20 (1.09, 1.33) p<0.001 | 1.46 (1.29, 1.66) p<0.001 | 2.57 (2.01, 3.30) p<0.001 |
| **Cognitive Impairment** | | | | |
| 6,684 (30.3%) | 2,392 (28%) | 2,726 (30%) | 1,253 (35%) | 313 (42%) |
| OR (95%CI) | 1 | 1.04 (0.96, 1.13) p=0.318 | 1.24 (1.12, 1.38) p<0.001 | 1.56 (1.28, 1.90) p<0.001 |
| FEV_1_/FVC >=LLN | 1 | 1.04 (0.96, 1.13) p=0.355 | 1.23 (1.10, 1.37) p<0.001 | 1.59 (1.28, 1.98) p<0.001 |
| **Gait speed, m/sec** | | | | |
| Mean 1.01 (sd 0.19) | 1.03 (0.18) | 1.01 (sd 0.19) | 0.98 (sd 0.20) | 0.93 (sd 0.19) |
| Difference to ref* | 0 | -0.008 (-0.014, -0.001)p=0.016 | -0.026 (-0.035, -0.017) p<0.001 | -0.063 (-0.079, -0.047) p<0.001 |
| FEV_1_/FVC >=LLN | 0 | -0.006 (-0.012, 0.001) p=0.081 | -0.025 (-0.034, -0.016) p<0.001 | -0.061 (-0.080, -0.043) p<0.001 |
| **Standing balance, sec** | | | | |
| Mean 45.4 (sd 21.3) | 47.9 (19.6) | 45.2 (sd 21.5) | 41.5 (sd 23.2) | 36.2 (sd 24.2) |
| Difference to ref* | 0 | -1.18 (-1.77, -0.58) p<0.001 | -3.25 (-4.11, -2.40) p<0.001 | -7.65 (-9.64, -5.66) p<0.001 |
| FEV_1_/FVC >=LLN | 0 | -1.20 (-1.81, -0.59) p<0.001 | -3.38 (-4.27, -2.49) p<0.001 | -7.54 (-9.76, -5.32) p<0.001 |
| **Timed Up and Go, sec** | | | | |
| Mean 9.2 (sd 2.1) | 9.0 (1.9) | 9.2 (sd 1.9) | 9.7 (sd 2.8) | 10.3 (sd 3.1) |
| Difference to ref* | 0 | 0.057 (-0.003, 0.117) p=0.065 | 0.360 (0.266, 0.455) p<0.001 | 0.833 (0.525, 1.141) p<0.001 |
| FEV_1_/FVC >=LLN | 0 | 0.051 (-0.011, 0.113) p=0.105 | 0.370 (0.271, 0.469) p<0.001 | 0.920 (0.555, 1.286) p<0.001 |
| **Grip strength, kg** | | | | |
| Mean 37.0 (sd 12.2) | 37.4 (12.0) | 36.8 (12.3) | 36.8 (12.3) | 35.2 (12.1) |
| Difference to ref* | 0 | -1.24 (-1.51, -0.97) p<0.001 | -2.59 (-2.96, -2.22) p<0.001 | -5.05 (-5.93, -4.17) p<0.001 |
| FEV_1_/FVC >=LLN | 0 | -1.24 (-1.51, -0.97) p<0.001 | -2.62 (-3.00, -2.24) p<0.001 | -5.29 (-6.27, -4.31) p<0.001 |

For each outcome, raw data expressed as frequencies or means (sd) for each FVC category are provided in the first row. Odds ratios (OR) or mean change with 95% CI and p-values were calculated for each FVC levels relative to reference (FVC >0sd) for the overall group in the second row. All estimates were adjusted for age, sex, BMI, smoking status (never, former, current); education (less than secondary, secondary, post-secondary); physical activity; self-reported asthma/ COPD/ cardiovascular disease; and the number of chronic conditions. Moderate-to-severe respiratory symptoms refers to breathlessness, cough or wheeze with walking on flat surfaces or occurring at night-time at least once per week. Analyses were performed for the overall cohort* and separately for the remaining participants (n=21,667) after removing those with FEV_1_/FVC < LLN or airflow obstruction.
