# Supplementary material for: Associations between lung function and physical and cognitive health in the Canadian Longitudinal Study on Aging (CLSA): A cross-sectional study from a multicenter national cohort
Source: PLoS Med. 2022 Feb 9;19(2):e1003909. doi: 10.1371/journal.pmed.1003909 (PMC8870596; doi:10.1371/journal.pmed.1003909)
Supplement: S4 Table — FEV1, forced expiratory volume in 1 second; SD, standard deviation. (DOCX) [file pmed.1003909.s006.docx]

**S4 Table**. Unadjusted stratified analysis by gender, smoking history and baseline age for self-perceived poor health, respiratory symptoms and low cognitive scores by grades of low FEV_1_ relative to reference group (FEV_1_ >0sd).

|  | **Categories of FEV_1_ according to GLI z-scores** | | | |
| --- | --- | --- | --- | --- |
|  | **>0sd** | **0 to >-1sd** | **-1 to >-2sd** | **=<-2sd** |
| Total 22,822 | Normal 8,626 | Mild 8,514 | Moderate 4,353 | Severe 1,329 |
| **Perceived poor health** | | | | |
| Males | 1 | 1.26 (1.02, 1.57) p=0.036 | 2.54 (2.03, 3.17) p<0.001 | 4.07 (3.06, 5.40) p<0.001 |
| Females | 1 | 1.40 (1.16, 1.70) p=0.001 | 2.02 (1.64, 2.49) p<0.001 | 4.06 (3.11, 5.31) p<0.001 |
|  | | | | |
| Smokers | 1 | 1.45 (1.19, 1.77) p<0.001 | 2.26 (1.84, 2.78) p<0.001 | 4.05 (3.17, 5.18) p<0.001 |
| Non-Smokers | 1 | 1.16 (0.94, 1.44) p=0.163 | 2.08 (1.65, 2.61) p<0.001 | 3.27 (2.33, 4.58) p<0.001 |
|  | | | | |
| 45-54 years | 1 | 1.39 (1.07, 1.79) p=0.012 | 2.08 (1.58, 2.74) p<0.001 | 4.19 (2.98, 5.87) p<0.001 |
| 55-64 | 1 | 1.24 (0.99, 1.56) p=0.061 | 2.33 (1.84, 2.95) p<0.001 | 3.72 (2.69, 5.16) p<0.001 |
| 65-74 | 1 | 1.42 (1.04, 1.92) p=0.025 | 3.01 (2.20, 4.12) p<0.001 | 5.09 (3.48, 7.45) p<0.001 |
| 75+ | 1 | 1.29 (0.93, 1.81) p=0.130 | 2.07 (1.46, 2.96) p<0.001 | 3.19 (2.02, 5.03) p<0.001 |
| **Moderate - Severe symptoms** | | | | |
| Males | 1 | 1.21 (1.05, 1.39) p=0.007 | 2.04 (1.74, 2.38) p<0.001 | 5.23 (4.14, 6.59) p<0.001 |
| Females | 1 | 1.46 (1.30, 1.64) p<0.001 | 2.09 (1.82, 2.40) p<0.001 | 5.19 (4.15, 6.48) p<0.001 |
|  | | | | |
| Smokers | 1 | 1.44 (1.27, 1.64) p<0.001 | 2.13 (1.84, 2.47) p<0.001 | 6.44 (5.20, 7.98) p<0.001 |
| Non-Smokers | 1 | 1.24 (1.10, 1.39) p<0.001 | 1.88 (1.63, 2.17) p<0.001 | 3.44 (2.68, 4.41) p<0.001 |
|  | | | | |
| 45-54 years | 1 | 1.21 (1.04, 1.42) p=0.016 | 1.78 (1.49, 2.14) p<0.001 | 4.49 (3.41, 5.91) p<0.001 |
| 55-64 | 1 | 1.37 (1.19, 1.58) p<0.001 | 2.13 (1.81, 2.50) p<0.001 | 5.50 (4.22, 7.17) p<0.001 |
| 65-74 | 1 | 1.49 (1.26, 1.77) p<0.001 | 2.68 (2.20, 3.28) p<0.001 | 6.29 (4.66, 8.49) p<0.001 |
| 75+ | 1 | 1.68 (1.36, 2.08) p<0.001 | 2.12 (1.65, 2.73) p<0.001 | 5.31 (3.56, 7.91) p<0.001 |
| **Cognitive Impairment** | | | | |
| Males | 1 | 1.04 (0.93, 1.16) p=0.503 | 1.15 (1.01, 1.32) p=0.034 | 1.39 (1.13, 1.71) p=0.002 |
| Females | 1 | 1.08 (0.98, 1.20) p=0.118 | 1.23 (1.08, 1.39) p=0.001 | 1.69 (1.39, 2.04) p<0.001 |
|  | | | | |
| Smokers | 1 | 1.11 (0.99, 1.24) p=0.076 | 1.21 (1.06, 1.38) p=0.004 | 1.36 (1.13, 1.63) p=0.001 |
| Non-Smokers | 1 | 1.02 (0.92, 1.13) p=0.654 | 1.15 (1.01, 1.31) p=0.029 | 1.77 (1.42, 2.21) p<0.001 |
|  | | | | |
| 45-54 years | 1 | 1.09 (0.95, 1.24) p=0.215 | 1.19 (1.02, 1.38) p=0.027 | 1.83 (1.44, 2.32) p<0.001 |
| 55-64 | 1 | 1.00 (0.88, 1.13) p=0.995 | 1.16 (1.00, 1.34) p=0.046 | 1.52 (1.21, 1.92) p<0.001 |
| 65-74 | 1 | 1.06 (0.91, 1.22) p=0.463 | 1.22 (1.02, 1.46) p=0.032 | 1.05 (0.79, 1.38) p=0.748 |
| 75+ | 1 | 1.20 (1.00, 1.45) p=0.056 | 1.22 (0.98, 1.53) p=0.078 | 1.22 (0.87, 1.70) p=0.246 |

Unadjusted Odds Ratios (OR), 95% CI and p-values were calculated relative to the reference group (FEV_1_ z score >0sd) using multi-level logistic regression. The regression model adjusted for levels of FEV_1_ as the only fixed covariate and center as random effect. Moderate-to-severe respiratory symptoms refers to breathlessness, cough or wheeze with walking on flat surfaces or occurring at night-time at least once per week.
