# Supplementary material for: Associations between lung function and physical and cognitive health in the Canadian Longitudinal Study on Aging (CLSA): A cross-sectional study from a multicenter national cohort
Source: PLoS Med. 2022 Feb 9;19(2):e1003909. doi: 10.1371/journal.pmed.1003909 (PMC8870596; doi:10.1371/journal.pmed.1003909)
Supplement: S5 Table — FEV1, forced expiratory volume in 1 second; SD, standard deviation. (DOCX) [file pmed.1003909.s007.docx]

**S5 Table.** Adjusted stratified analyses by gender, smoking history and baseline age for self-perceived poor health, respiratory symptoms and low cognitive scores by grades of low FEV_1_ relative to reference group (FEV_1_ >0sd).

|  | **Categories of FEV_1_ according to GLI z-scores** | | | |
| --- | --- | --- | --- | --- |
|  | **>0sd** | **0 to >-1sd** | **-1 to >-2sd** | **=<-2sd** |
| Total 22,822 | Normal 8,626 | Mild 8,514 | Moderate 4,353 | Severe 1,329 |
| **Perceived poor health** | | | | |
| Males | 1 | 1.02 (0.79, 1.31) p=0.889 | 1.75 (1.35, 2.27) p<0.001 | 1.81 (1.26, 2.60) p=0.001 |
| Females | 1 | 1.13 (0.89, 1.42) p=0.309 | 1.27 (0.98, 1.63) p=0.068 | 1.84 (1.31, 2.58) p<0.001 |
|  | | | | |
| Smokers | 1 | 1.26 (0.99, 1.58) p=0.054 | 1.63 (1.28, 2.08) p<0.001 | 2.29 (1.67, 3.15) p<0.001 |
| Non-Smokers | 1 | 0.91 (0.70, 1.16) p=0.436 | 1.45 (1.11, 1.90) p=0.006 | 1.60 (1.08, 2.37) p=0.019 |
|  | | | | |
| 45-54 years | 1 | 1.12 (0.83, 1.52) p=0.443 | 1.56 (1.14, 2.12) p=0.005 | 1.66 (1.09, 2.55) p=0.019 |
| 55-64 | 1 | 0.98 (0.76, 1.29) p=0.920 | 1.32 (0.99, 1.96) p=0.059 | 1.60 (1.05, 2.44) p=0.029 |
| 65-74 | 1 | 1.15 (0.81, 1.63) p=0.429 | 1.77 (1.20, 2.61) p=0.004 | 2.83 (1.75, 4.59) p<0.001 |
| 75+ | 1 | 1.03 (0.68, 1.57) p=0.885 | 1.43 (0.90, 2.27) p=0.127 | 2.15 (1.18, 3.91) p=0.012 |
| **Moderate - Severe symptoms** | | | | |
| Males | 1 | 0.94 (0.81, 1.09) p=0.418 | 1.44 (1.20, 1.71) p<0.001 | 2.63 (2.01, 3.45) p<0.001 |
| Females | 1 | 1.22 (1.07, 1.39) p=0.003 | 1.44 (1.22, 1.69) p<0.001 | 2.67 (2.05, 3.48) p<0.001 |
|  | | | | |
| Smokers | 1 | 1.22 (1.05, 1.41) p=0.007 | 1.64 (1.39, 1.94) p<0.001 | 3.96 (3.04, 5.14) p<0.001 |
| Non-Smokers | 1 | 1.02 (0.89, 1.17) p=0.747 | 1.39 (1.18, 1.64) p<0.001 | 2.03 (1.52, 2.71) p<0.001 |
|  | | | | |
| 45-54 years old | 1 | 1.01 (0.85, 1.20) p=0.914 | 1.35 (1.10, 1.66) p=0.004 | 2.31 (1.67, 3.21) p<0.001 |
| 55-64 | 1 | 1.08 (0.92, 1.26) p=0.341 | 1.46 (1.21, 1.77) p<0.001 | 2.71 (2.01, 3.67) p<0.001 |
| 65-74 | 1 | 1.19 (0.99, 1.45) p=0.068 | 1.84 (1.46, 2.31) p<0.001 | 3.34 (2.31, 4.83) p<0.001 |
| 75+ | 1 | 1.37 (1.07, 1.76) p=0.012 | 1.23 (0.89, 1.69) p=0.207 | 3.47 (2.09, 5.76) p<0.001 |
| **Cognitive Impairment** | | | | |
| Males | 1 | 0.97 (0.86, 1.10) p=0.640 | 1.06 (0.91, 1.22) p=0.443 | 1.24 (0.98, 1.47) p=0.069 |
| Females | 1 | 1.09 (0.97, 1.22) p=0.124 | 1.25 (1.09, 1.43) p=0.002 | 1.55 (1.24, 1.94) p<0.001 |
|  | | | | |
| Smokers | 1 | 1.09 (0.96, 1.23) p=0.188 | 1.20 (1.04, 1.38) p=0.015 | 1.30 (1.05, 1.61) p=0.016 |
| Non-Smokers | 1 | 1.00 (0.90, 1.12) p=0.896 | 1.15 (0.99, 1.32) p=0.051 | 1.67 (1.31, 2.14) p<0.001 |
|  | | | | |
| 45-54 years old | 1 | 1.05 (0.91, 1.21) p=0.526 | 1.17 (0.99, 1.38) p=0.072 | 1.63 (1.25, 2.13) p<0.001 |
| 55-64 | 1 | 0.96 (0.84, 1.10) p=0.575 | 1.09 (0.93, 1.29) p=0.282 | 1.30 (0.99, 1.71) p=0.055 |
| 65-74 | 1 | 1.02 (0.87, 1.21) p=0.777 | 1.22 (0.99, 1.49) p=0.050 | 0.99 (0.71, 1.37) p=0.938 |
| 75+ | 1 | 1.24 (1.00, 1.52) p=0.045 | 1.15 (0.89, 1.50) p=0.273 | 1.25 (0.83, 1.87) p=0.291 |

Adjusted Odds Ratios (OR), 95% CI and p-values were calculated relative to reference group (FEV_1_ z score >0sd) using multi-level logistic regression adjusted for age, sex, BMI, smoking status (never, former, current); education (less than secondary, secondary, post-secondary); physical activity; self-reported asthma/COPD/ cardiovascular disease; and number of chronic conditions. Moderate-to-severe respiratory symptoms refers to breathlessness, cough or wheeze with walking on flat surfaces or occurring at night-time at least once per week.
