# Supplementary material for: Associations between lung function and physical and cognitive health in the Canadian Longitudinal Study on Aging (CLSA): A cross-sectional study from a multicenter national cohort
Source: PLoS Med. 2022 Feb 9;19(2):e1003909. doi: 10.1371/journal.pmed.1003909 (PMC8870596; doi:10.1371/journal.pmed.1003909)
Supplement: S6 Table — FEV1, forced expiratory volume in 1 second; SD, standard deviation. (DOCX) [file pmed.1003909.s008.docx]

**S6 Table.** Unadjusted stratified analyses by gender, smoking history and baseline age groups on physical performance according to grades of low FEV_1_ relative to reference group (FEV_1_ >0sd).

|  | **Categories of FEV_1_ according to GLI z-scores** | | | |
| --- | --- | --- | --- | --- |
|  | **>0sd** | **0 to >-1sd** | **-1 to >-2sd** | **=<-2sd** |
| Total 22,822 | 8,626 | Mild 8,514 | Moderate 4,353 | Severe 1,329 |
| **Gait speed, m/sec** | | | | |
| Males | 0 | -0.007 (-0.015, 0.02) p=0.144 | -0.014 (-0.025, -0.003) p=0.014 | -0.063 (-0.079, -0.046) p<0.001 |
| Females | 0 | -0.015 (-0.024, -0.006) p=0.002 | -0.055 (-0.066, -0.044) p<0.001 | -0.087 (-0.105, -0.069) p<0.001 |
|  | | | | |
| Smokers | 0 | -0.011 (-0.020, -0.001) p=0.025 | -0.036 (-0.047, -0.025) p<0.001 | -0.071 (-0.087, -0.054) p<0.001 |
| Non-Smokers | 0 | -0.003 (-0.012, 0.006) p=0.487 | -0.017 (-0.029, -0.006) p=0.002 | -0.054 (-0.074, -0.034) p<0.001 |
|  | | | | |
| 45-54 years | 0 | -0.002 (-0.013, 0.009) p=0.733 | -0.019 (-0.033, -0.006) p=0.004 | -0.061 (-0.081, -0.041) p<0.001 |
| 55-64 | 0 | -0.012 (-0.048, -0.002) p=0.020 | -0.036 (-0.048, -0.023) p<0.001 | -0.088 (-0.109, -0.066) p<0.001 |
| 65-74 | 0 | -0.017 (-0.029, -0.004) p=0.007 | -0.056(-0.070, -0.041) p<0.001 | -0.076 (-0.101, -0.051) p<0.001 |
| 75+ | 0 | -0.036 (-0.052, -0.021) p<0.001 | -0.062 (-0.080, -0.043) p<0.001 | -0.072 (-0.100, -0.044) p<0.001 |
| **Standing balance, sec** | | | | |
| Males | 0 | -2.16 (-3.12, -1.20) p<0.001 | -3.95 (-5.16, -2.74) p<0.001 | -8.53 (-10.65, -6.41) p<0.001 |
| Females | 0 | -2.27 (-3.21, -1.33) p<0.001 | -5.74 (-7.01, -4.48) p<0.001 | -10.51 (-12.71, -8.31) p<0.001 |
|  | | | | |
| Smokers | 0 | -2.37 (-3.43, -1.31) p<0.001 | -6.18 (-7.52, -4.85) p<0.001 | -10.95 (-12.97, -8.92) p<0.001 |
| Non-Smokers | 0 | -1.95 (-2.82, -1.08) p<0.001 | -3.04 (-4.20, -1.89) p<0.001 | -6.27 (-8.61, -3.93) p<0.001 |
|  | | | | |
| 45-54 years | 0 | -0.96 (-1.88, -0.04) p=0.041 | -2.96 (-4.18, -0.04) p<0.001 | -6.79 (-9.02, -4.56) p<0.001 |
| 55-64 | 0 | -3.35 (-4.41, -2.28) p<0.001 | -6.21 (-7.60, -4.82) p<0.001 | -11.06 (-13.61, -8.52) p<0.001 |
| 65-74 | 0 | -3.42 (-4.91, -1.92) p<0.001 | -7.66 (-9.54, -5.79) p<0.001 | -12.81 (-15.65, -9.97) p<0.001 |
| 75+ | 0 | -2.17 (-3.88, -0.46) p=0.013 | -5.81 (-7.73, -3.88) p<0.001 | -7.65 (-10.27, -5.04) p<0.001 |
| **Timed up and go, sec** | | | | |
| Males | 0 | 0.08 (-0.01, 0.17) p=0.097 | 0.35 (0.22, 0.47) p<0.001 | 0.91 (0.61, 1.21) p<0.001 |
| Females | 0 | 0.17 (0.09, 0.25) p<0.001 | 0.58 (0.47, 0.70) p<0.001 | 0.98 (0.76, 1.20) p<0.001 |
|  | | | | |
| Smokers | 0 | 0.19 (0.10, 0.28) p<0.001 | 0.57 (0.44, 0.70) p<0.001 | 1.11 (0.83, 1.39) p<0.001 |
| Non-Smokers | 0 | 0.06 (-0.02, 0.14) p=0.128 | 0.34 (0.23, 0.45) p<0.001 | 0.63 (0.42, 0.83) p<0.001 |
|  | | | | |
| 45-54 years | 0 | 0.02 (-0.07, 0.12) p=0.640 | 0.27 (0.13, 0.40) p<0.001 | 0.67 (0.33, 1.00) p<0.001 |
| 55-64 | 0 | 0.17 (0.07, 0.27) p=0.001 | 0.47 (0.34, 0.59) p<0.001 | 0.99 (0.77, 1.20) p<0.001 |
| 65-74 | 0 | 0.16 (0.04, 0.28) p=0.009 | 0.79 (0.60, 0.98) p<0.001 | 1.11 (0.78, 1.44) p<0.001 |
| 75+ | 0 | 0.42 (0.19, 0.66) p<0.001 | 0.94 (0.64, 1.24) p<0.001 | 1.54 (0.93, 2.15) p<0.001 |
| **Grip strength, kg** | | | | |
| Males | 0 | -1.38 (-1.87, -0.90) p<0.001 | -2.48 (-3.06, -1.89) p<0.001 | -4.64 (-5.62, -3.65) p<0.001 |
| Females | 0 | -0.79 (-1.08, -0.51) p<0.001 | -1.53 (-1.89, -1.18) p<0.001 | -2.85 (-3.44, -2.27) p<0.001 |
|  | | | | |
| Smokers | 0 | -0.42 (-1.08, 0.23) p=0.207 | -1.41 (-2.16, -0.66) p<0.001 | -2.16 (-3.28, -1.04) p<0.001 |
| Non-smokers | 0 | -1.33 (-1.92, -0.73) p<0.001 | -1.03 (-1.81, -0.25) p=0.009 | -3.56 (-4.93, -2.18) p<0.001 |
|  | | | | |
| 45-54 years | 0 | -1.01 (-1.78, -0.23) p=0.011 | -0.95 (-1.87, -0.02) p=0.044 | -2.84 (-4.34, -1.33) p<0.001 |
| 55-64 | 0 | -1.05 (-1.70, -0.39) p=0.002 | -1.55 (-2.35, -0.74) p<0.001 | -3.96 (-5.28, -2.63) p<0.001 |
| 65-74 | 0 | -0.62 (-1.35, 0.11) p=0.096 | -1.68 (-2.58, -0.78) p<0.001 | -0.97 (-2.33, 0.39) p<0.001 |
| 75+ | 0 | -1.00 (-1.85, -0.14) p=0.022 | -1.50 (-2.49, -0.52) p=0.003 | -1.58 (-3.14, -0.02) p=0.047 |

Estimates of mean differences, 95%CI and p-values in physical performance relative to the reference group (FEV_1_%>0sd) within stratum were unadjusted using multi-level linear regression adjusting for levels of FEV_1_ as the only fixed covariate and center as random effect.
