# Supplementary material for: Associations between lung function and physical and cognitive health in the Canadian Longitudinal Study on Aging (CLSA): A cross-sectional study from a multicenter national cohort
Source: PLoS Med. 2022 Feb 9;19(2):e1003909. doi: 10.1371/journal.pmed.1003909 (PMC8870596; doi:10.1371/journal.pmed.1003909)
Supplement: S7 Table — FEV1, forced expiratory volume in 1 second; SD, standard deviation. (DOCX) [file pmed.1003909.s009.docx]

**S7 Table.** Adjusted stratified analyses by gender, smoking history and baseline age groups on physical performance according to grades of lower FEV_1_ z scores relative to reference group (FEV_1_ >0sd).

|  | **Categories of FEV_1_ according to GLI z-scores** | | | |
| --- | --- | --- | --- | --- |
|  | **>0sd** | **0 to >-1sd** | **-1 to >-2sd** | **=<-2sd** |
| Total 22,822 | 8,626 | Mild 8,514 | Moderate 4,353 | Severe 1,329 |
| **Gait speed, m/sec** | | | | |
| Males | 0 | 0.001 (-0.008, 0.010) p=0.821 | 0.001 (-0.011, 0.012) p=0.931 | -0.031 (-0.049, -0.012) p=0.001 |
| Females | 0 | -0.005 (-0.014, 0.004) p=0.256 | -0.036 (-0.047, -0.025) p<0.001 | -0.049 (-0.068, -0.029) p<0.001 |
|  | | | | |
| Smokers | 0 | -0.002 (-0.012, 0.008) p=0.686 | -0.020 (-0.031, -0.009) p=0.001 | -0.034 (-0.053, -0.016) p<0.001 |
| Non-Smokers | 0 | -0.003 (-0.012, 0.006) p=0.487 | -0.017 (-0.029, -0.006) p=0.002 | -0.054 (-0.074, -0.034) p<0.001 |
|  | | | | |
| 45-54 years | 0 | 0.005 (-0.006, 0.016), p=0.378 | -0.009 (-0.023, 0.005) p=0.191 | -0.03 (-0.05, -0.008) p=0.008 |
| 55-64 | 0 | -0.004 (-0.014, 0.008) p=0.509 | -0.015 (-0.028, -0.002) p=0.025 | -0.06 (-0.082, -0.035) p<0.001 |
| 65-74 | 0 | -0.006 (-0.019, 0.006) p=0.334 | -0.032 (-0.047, -0.017) p<0.001 | -0.032 (-0.06, -0.004) p=0.023 |
| 75+ | 0 | -0.025 (-0.041, -0.009) p=0.002 | -0.039 (-0.059, -0.019) p<0.001 | -0.048 (-0.080, -0.016) p=0.003 |
| **Standing balance, sec** | | | | |
| Males | 0 | -0.94 (-1.85, -0.04) p=0.042 | -1.85 (-2.97, -0.72) p=0.001 | -4.21 (-6.23, -2.18) p<0.001 |
| Females | 0 | -1.02 (-1.87, -0.18) p=0.018 | -3.33 (-4.47, -2.19) p<0.001 | -6.51 (-8.56, -4.47) p<0.001 |
|  | | | | |
| Smokers | 0 | -0.62 (-1.60, 0.36) p=0.217 | -3.67 (-4.93, -2.42) p<0.001 | -5.86 (-7.79, -3.92) p<0.001 |
| Non-Smokers | 0 | -1.36 (-2.15, -0.56) p=0.001 | -1.88 (-2.92, -0.84) p<0.001 | -5.60 (-7.79, -3.41) p<0.001 |
|  | | | | |
| 45-54 years | 0 | -0.24 (-1.19, 0.72) p=0.626 | -1.24 (-2.47, -0.000) p=0.050 | -3.63 (-5.92, -1.34) p=0.002 |
| 55-64 | 0 | -1.65 (-2.74, -0.57) p=0.003 | -3.34 (-4.78, -1.90) p<0.001 | -6.50 (-9.12, -3.88) p<0.001 |
| 65-74 | 0 | -1.58 (-3.10, -0.05) p=0.042 | -3.86 (-5.82, -1.91) p<0.001 | -7.48 (-10.73, -4.23) p<0.001 |
| 75+ | 0 | -0.64 (-2.47, 1.18) p=0.488 | -4.26 (-6.41, -2.10) p<0.001 | -7.16 (-10.41, -3.89) p<0.001 |
| **Timed up and go, sec** | | | | |
| Males | 0 | -0.015 (-0.109, 0.08) p=0.758 | 0.19 (0.06, 0.31) p=0.004 | 0.61 (0.26, 0.96) p=0.001 |
| Females | 0 | 0.08 (-0.003, 0.16) p=0.059 | 0.36 (0.25, 0.48) p<0.001 | 0.40 (0.23, 0.57) p<0.001 |
|  | | | | |
| Smokers | 0 | 0.07 (-0.023, 0.168) p=0.138 | 0.35 (0.21, 0.49) p<0.001 | 0.62 (0.27, 0.96) p<0.001 |
| Non-Smokers | 0 | 0.014 (-0.067, 0.09) p=0.736 | 0.24 (0.13, 0.35) p<0.001 | 0.46 (0.26, 0.66) p<0.001 |
|  | | | | |
| 45-54 years | 0 | -0.04 (-0.14, 0.06) p=0.405 | 0.15 (0.005, 0.289) p=0.043 | 0.365 (-0.021, 0.751) p=0.064 |
| 55-64 | 0 | 0.082 (-0.025, 0.190) p=0.132 | 0.248 (0.110, 0.386) p<0.001 | 0.621 (0.404, 0.837) p<0.001 |
| 65-74 | 0 | 0.029 (-0.097, 0.154) p=0.653 | 0.474 (0.291, 0.658) p<0.001 | 0.447 (0.156, 0.737) p=0.003 |
| 75+ | 0 | 0.229 (0.008, 0.450) p=0.042 | 0.643 (0.319, 0.967) p<0.001 | 1.126 (0.621, 1.630) p<0.001 |
| **Grip strength, kg** | | | | |
| Males | 0 | -1.42 (-1.90, -0.94) p<0.001 | -2.44 (-3.04, -1.84) p<0.001 | -4.18 (-5.26, -3.09) p<0.001 |
| Females | 0 | -0.80 (-1.07, -0.52) p<0.001 | -1.42 (-1.77, -1.06) p<0.001 | -2.68 (-3.30, -2.06) p<0.001 |
|  | | | | |
| Smokers | 0 | -0.69 (-1.11, -0.27) p=0.001 | -1.53 (-2.01, -1.04) p<0.001 | -2.54 (-3.40, -1.67) p<0.001 |
| Non-Smokers | 0 | -1.34 (-1.71, -0.98) p<0.001 | -2.14 (-2.64, -1.64) p<0.001 | -4.41 (-5.38, -3.44) p<0.001 |
|  | | | | |
| 45-54 years | 0 | -1.12 (-1.615, -0.624) p<0.001 | -2.23 (-2.85, -1.61) p<0.001 | -3.77 (-4.92, -2.63) p<0.001 |
| 55-64 | 0 | -1.18 (-1.60, -0.75) p<0.001 | -1.59 (-2.14, -1.04) p<0.001 | -4.00 (-4.96, -3.05) p<0.001 |
| 65-74 | 0 | -0.63 (-1.10, -0.15) p=0.010 | -1.62 (-2.22, -1.02) p<0.001 | -2.23 (-3.28, -1.17) p<0.001 |
| 75+ | 0 | -1.33 (-1.92, -0.74) p<0.001 | -1.89 (-2.62, -1.16) p<0.001 | -2.52 (-3.66, -1.38) p<0.001 |

Multilevel linear regression was used to estimate the mean differences (and 95%CI) in physical performance relative to reference (FEV_1_%>0sd) within stratum. Models adjust for age (omitted for age stratified analysis), sex (omitted for sex stratified analysis), BMI, smoking status (omitted for smoking stratified analysis); education; physical activity, self-reported asthma/ COPD/CVD and number of self-reported chronic non-communicable conditions.
