# Supplementary material for: Associations between lung function and physical and cognitive health in the Canadian Longitudinal Study on Aging (CLSA): A cross-sectional study from a multicenter national cohort
Source: PLoS Med. 2022 Feb 9;19(2):e1003909. doi: 10.1371/journal.pmed.1003909 (PMC8870596; doi:10.1371/journal.pmed.1003909)
Supplement: Attachment — Submitted filename: Reviewer_responseNov2021.1.docx [file pmed.1003909.s010.docx]

Requests from the editors:

From the Academic Editor:

1. I agree with one of the reviewers that this is an association study. The use of 'effect' suggests causality. This should be attended to.

- We have carefully revised the title and text of the manuscript to avoid implicating an effect or causality.

2. The focus is on FEV1. it would be nice to get some more information on the standardisation procedures for its measurement. This is a notorious measure in the field, especially if it is being done by several different research assistants (which is likely to be the case). There are some pre-requirements - not having smoked for an hour, not after a large meal, avoid alcohol, etc. It is best done in the office, but field measurement may have been the only option. When compared to normative data, they had 25% <1 SD and 6% <2 SD, which suggests a sample skewed to the left (although this is a large population-based study). This does not negate the results, but is worthy of discussion.

- The training and conduct of spirometry was standardized across sites. Even though, we did not specify these pre-requirements, in practice, they were met since all participants underwent the same schedule of tests (physical measurements, ECG, carotid ultrasound) which took approximately 45-60 minutes to complete before undertaking spirometry. During this time, participants would not be able to eat large meals, smoke/vape or exercise for 45 minutes or more. Participants were also encouraged to wear loose clothing to make it easier to take these measurements and perform spirometry. All tests/ visits were conducted in dedicated research clinics under controlled and standardized temperature and ventilation conditions. This information has been added to the methods section.
- This is a randomly selected sample of the Canadian general population from predefined geographic areas of adults between the ages 45-85 at the time of recruitment across 11 sites and 7 provinces. We have compared our population to the Canadian census data to assess its generalizability (<https://academic.oup.com/ije/article/48/6/1752/5563992>) The distribution of participants according to z-score were 38% no impairment (FEV_1_>0sd); 37% (n=8,514) mildly low (0 to >-1sd); 19% (n=4,353) moderately low (-1sd to >-2sd); and 6% (n=1,329) severely low FEV_1_ (=<-2sd). There is a slight skewness of the data to the left suggesting inclusion of more individuals with severe FEV_1_ impairment. The higher proportion of individuals below the LLN (<2sd) has also been shown in other populations from high income countries, where the demographic is of an aging population living with higher prevalence of chronic diseases including chronic lung diseases. In a recent report using the NHANES dataset, the proportion of individuals with GLI FEV1 and FVC z scores <-2SD were 8.2% and 9.9% respectively (Luoto J et al, Eur Respir J 2019; 53).

3. There are several measures of frailty that are being linked to cognition and to each other. A multivariate regression model may be instructive to see if FEV1 has any predictive value over and above the other measures? Is it a unique association, or is it that since frailty is known to be linked to cognition, and the measures of frailty are correlated, and we are seeing this in a univariate analysis.

The present study was aimed to explore the association between lung function with boarder markers of general health, which may have potential implications on predicting the aging trajectory. We have selected few indicators of health that may or may not be included in the definition of frailty depending on which definition of frailty one chooses. We were not looking at frailty, but were looking at health, physical function and cognition. Given these findings, we plan in future analyses to explore these association with cumulative deficit model of frailty as it does not include FEV1 as one of the indicators of frailty.

4. Do they have any other measure of general health? Many studies do use self-report on one question, but it would be nice to have something more comprehensive and/or objective.

- We do not have any other marker of general health. However, over two decade of research on the self-perceived global (SPH) measure of health status has shown that SPH is a robust measure of health status and independently predict mortality, hospitalization and poor functional outcomes after controlling comprehensively for physician diagnosis, self-reported chronic conditions, other clinical and laboratory related health data. The fact that a large number of studies using different study design, cohort demographics, covariates adjustments suggest that it is a robust marker for poor outcomes with high predictive value.

Additional editorial requests:

5. Please revise your title according to PLOS Medicine's style. Your title must be nondeclarative and not a question. It should begin with main concept if possible. "Effect of" should be used only if causality can be inferred, i.e., for an RCT. Please place the study design ("A randomized controlled trial," "A retrospective study," "A modelling study," etc.) in the subtitle (ie, after a colon).

- The title has been changed to “Associations between lung function with physical and cognitive health in the Canadian Longitudinal Study on Aging (CLSA): a multi-center population-based observational cohort study.

6. Financial disclosure: Please describe the sources of funding for the study, in addition to noting that The funders had no role in study design, data collection and analysis, decision to publish, or preparation of the manuscript.

- The funding information has been added.

  7. Data availability statement: The Data Availability Statement (DAS) requires revision. PLOS Medicine requires that the de-identified data underlying the specific results in a published article be made available, without restrictions on access, in a public repository or as Supporting Information at the time of article publication, provided it is legal and ethical to do so. Please see the policy at

  <http://journals.plos.org/plosmedicine/s/data-availability>

  and FAQs at

  <http://journals.plos.org/plosmedicine/s/data-availability#loc-faqs-for-data-policy>

  For each data source used in your study:

  a) If the data are freely or publicly available, note this and state the location of the data: within the paper, in Supporting Information files, or in a public repository (include the DOI or accession number).
- The CLSA data is freely available to all qualified researchers. The details for data access application are provided in the manuscript.

  b) If the data are owned by a third party but freely available upon request, please note this and state the owner of the data set and contact information for data requests (web or email address). Note that a study author cannot be the contact person for the data.

  c) If the data are not freely available, please describe briefly the ethical, legal, or contractual restriction that prevents you from sharing it. Please also include an appropriate contact (web or email address) for inquiries (again, this cannot be a study author).

  8. Throughout: Please include line numbers with the revised version.
- Line numbers have been inserted throughout the manuscript.

9. Abstract: Please structure your abstract using the PLOS Medicine headings (Background, Methods and Findings, Conclusions). Please combine the Methods and Findings sections into one section, “Methods and findings”.

- The abstract has been revised accordingly.

10. Abstract: Background: Abstract Background: The final sentence should clearly state the study question.

- The last sentence of the abstract background now reads ‘The present study seeks to address the association between forced expiratory volume in 1 second (FEV_1_) with broad markers of general health, relevant to the aging trajectory in the general population.’

11. Abstract: Methods and Findings: * Please include the study design, population and setting, number of participants, years during which the study took place, length of follow up, and main outcome measures. * Please quantify the main results (with 95% CIs and p values). * In the last sentence of the Abstract Methods and Findings section, please describe the main limitation(s) of the study's methodology.

- The abstract has been revised to include these data.

12. Abstract: Conclusions: * Please address the study implications without overreaching what can be concluded from the data; the phrase "In this study, we observed ..." may be useful.

- The conclusion has been revised to ‘We observed a robust and graded association between lower FEV_1_ with higher odds of respiratory symptoms, poor general health rating, lower cognitive and physical performance. These findings support the boarder implications of measured lung function on general health and the trajectory of aging.’

13. Author summary: At this stage, we ask that you include a short, non-technical Author Summary of your research to make findings accessible to a wide audience that includes both scientists and non-scientists. The Author Summary should immediately follow the Abstract in your revised manuscript. This text is subject to editorial change and should be distinct from the scientific abstract. Please see our author guidelines for more information: <https://journals.plos.org/plosmedicine/s/revising-your-manuscript#loc-author-summary>

- We have added the Author Summary (see below) following the abstract.

**AUTHOR SUMMARY:**

**Why was the study done?**

- Lung capacity is a measurable marker of lung health.
- Low lung capacity has been strongly linked to higher risks of death and poor heart and lung outcomes.
- Little is known on the broader association with general health, cognitive and physical function.

**What did the researchers do and find?**

- From a large general Canadian cohort study, 22,822 adults (52% females, average age 58.8 years) from 11 Canadian cities and 7 provinces, completed an interview-based questionnaire, spirometry, cognitive and physical performance testing.
- We observed a graded association between lower lung function with higher likelihood of moderate to severe breathlessness, poor general health rating, and low cognitive performance. Similar graded associations were also seen between lower lung function with lower performance on mobility, balance and strength.
- These associations exist throughout the range of lung function values even at mild levels considered within the normal range. Furthermore, they were consistent across groups of different ages, sex, smoking status and different patterns of lung function impairment.

**What do these findings mean?**

- These findings suggest that lung function may provide important information on general health and likely future cognitive and physical outcomes in the general population.

14. Throughout: For in-text reference citations, please place reference numbers in square brackets before the sentence punctuation, like this [1]. For multiple references, please do not include spaces within brackets.

- This has been revised accordingly throughout the manuscript.

15. Methods: Please provide the name(s) of the institutional review board(s) that provided ethical approval for the study, and please specify whether informed consent of participants was written or oral.

- This has been added and revised accordingly in the METHODS section.

16. Methods: Please provide more clarification on the selection of/ any differences between those selected for physical/cognitive/clinical assessments vs the tracking cohort. “Selected participants
were interviewed and had standardized physical, cognitive and clinical assessments
(comprehensive cohort) to provide data on demographics, lifestyle, health, and clinical
information. In the remaining participants (tracking cohort, n=21,241), similar data was collected
by a telephone interview”

- Participants in the comprehensive cohort was a random sample selected within 50km of each data collecting site/ dedicated research clinic. We have added a baseline characteristic table in the Appendix 2 (see below) for the comprehensive and tracking cohorts to allow comparison between the 2 groups within the CLSA
- Baseline Characteristics of the comprehensive and tracking cohorts.

|  | **Comprehensive cohort** | **Tracking cohort** |
| --- | --- | --- |
| N, % | 22,822, (100%) | 21,241 (100%) |
| Female | 11,981 (52%) | 10,835 (52%) |
| Age, yrs 45-54 | 6,235 (44%) | 5,832 (36%) |
| 55-64 | 7,769 (30%) | 6,564 (31%) |
| 65-74 | 5,396 (16%) | 4,634 (20%) |
| 75+ | 3,422 (10%) | 4,211 (13%) |
| Height, m (sd) | 1.69 (sd 0.1) | 1.70 (sd 0.3) |
| BMI, kgm^-2^ <25 | 7,082 (33%) | 7,758 (38%) |
| 25-30 | 9,226 (40%) | 8,232 (38%) |
| >30 | 6,496 (26%) | 5,142 (24%) |
| Education Primary | 1,074 (4%) | 1,986 (8%) |
| Secondary/Trade | 2,114 (9%) | 2,882 (14%) |
| University | 19,602 (87%) | 16,290 (78%) |
| Smoking Never | 7,318 (34%) | 6,239 (31%) |
| Former | 13,515 (57%) | 12,630 (58%) |
| Current | 1,851 (8%) | 2,267 (11%) |
| Physical activity | 153.2 (sd 76.6) | 151.2 (sd 78.8) |
| COPD | 1,040 (4%) | 1,436 (5.7%) |
| Asthma | 2,940 (13%) | 2,347 (11.3%) |
| CVD | 2,593 (9%) | 2,925 (11.7%) |
| No chronic conditions | 3,297 (20%) | 3,214 (18%) |
| >=3 chronic conditions | 8,664 (35%) | 8,736 (38%) |

Data are provided as frequencies (% of total column) for categorical variables or means (sd) for continuous variables for continuous variables. BMI= body mass index calculated as weight (kg) divided by height (m) squared; physical activity score was calculated using the PASE instrument with higher scores indicating higher physical activity; asthma, COPD (chronic obstructive pulmonary disease), CVD (cardiovascular disease) and chronic conditions were all obtained from self-reported interview-based questionnaires at baseline.

17. Methods: Please clarify: “Participants screened positive for major contra-indications were
Excluded.”

- We have revised this line to state “Participants screened positive for any major contra-indications (Appendix 3) to spirometry were excluded.”
- The list of contraindications is added in Appendix 3.

**Contraindications to performing spirometry:**

Severe acute respiratory condition

Pregnancy (over 27 weeks)

Unstable heart disease

Heart surgery within the last 3 months

Major surgery on chest or abdomen within the last 3 months

Detached retina or recent eye surgery in the last 3 months

Blood in sputum in last 3 months

Thoracic, abdominal or cerebral aneurysm(s) present

Average blood pressure of 200/120 or above (Blood Pressure taken at research clinic).

18. Methods: Please remove the trademark symbol from (Tracker Freedom®Wireless).

- This has been removed.

19. Methods: Did your study have a prospective protocol or analysis plan? Please state this (either way) early in the Methods section.

- We have added this statement to the METHODS section ‘A protocol of the planned analysis (Appendix 1) was submitted to the CLSA Data and Sample Access Committee and the Hamilton Health Sciences Ethnics Committee for approval prior to accessing the data and analysis.’

a) If a prospective analysis plan (from your funding proposal, IRB or other ethics committee submission, study protocol, or other planning document written before analyzing the data) was used in designing the study, please include the relevant prospectively written document with your revised manuscript as a Supporting Information file to be published alongside your study, and cite it in the Methods section. A legend for this file should be included at the end of your manuscript.

- The analysis plan and proposal which has been approved by ethics committee has been added to the supplemental material. Any modifications to the protocol has also been highlighted.

  b) If no such document exists, please make sure that the Methods section transparently describes when analyses were planned, and when/why any data-driven changes to analyses took place.

  c) In either case, changes in the analysis-- including those made in response to peer review comments-- should be identified as such in the Methods section of the paper, with rationale.

20. Methods: Please ensure that the study is reported according to the STROBE guideline, and include the completed STROBE checklist as Supporting Information. Please add the following statement, or similar, to the Methods: "This study is reported as per the Strengthening the Reporting of Observational Studies in Epidemiology (STROBE) guideline (S1 Checklist)."

The STROBE guideline can be found here: <http://www.equator-network.org/reporting-guidelines/strobe/>

When completing the checklist, please use section and paragraph numbers, rather than page numbers.

- Completed as suggested above

  21. Results: Throughout, please present the main results in the text, with 95% CIs and p values. For the assessment of respiratory, health, cognitive status with FEV1, please report both the adjusted and unadjusted analyses (with 95% CIs and p values).
- The RESULT section has been revised accordingly.

22. Results: Please provide quantitative results for these findings (reported with 95% CIs and p values): “The proportion of obstructive impairment (whether defined by FEV1/FVC <0.70 or <LLN), increased with lower FEV1; reaching approximately 50% in the severely low FEV1 group. The distribution of age, sex, and height across the FEV1 categories were similar. However, lower FEV1 was associated with lower physical activity, lower education level (primary school or lower); and higher rates of current smoking, obesity (BMI >30 kg/m2), asthma, COPD, CVD and multiple co-morbidities.”

- This section/ paragraph has been revised accordingly.

  23. Discussion: Please present and organize the Discussion as follows: a short, clear summary of the article's findings; what the study adds to existing research and where and why the results may differ from previous research; strengths and limitations of the study; implications and next steps for research, clinical practice, and/or public policy; one-paragraph conclusion.
- The DISCUSSION section has been revised and structured as suggested.

24. Discussion: We suggest tempering this statement, as a formal dose-response analysis did not seem to be reported: “The novel finding here, is the highly consistent dose-response relationship between lower FEV1 with higher prevalence and ORs of self-reported respiratory symptoms…”

- This sentence has been changed to “The novel finding here, is the highly consistent graded association between lower FEV1 with higher prevalence and ORs of self-reported respiratory symptoms…”

25. Table 2 and Table 3: Please provide the exact p values rather than p<0.05, etc. Please report p<0.001 where applicable. Please also provide the results from unadjusted analyses.

- This has been revised as suggested with p-values provided.
- The unadjusted analyses have been added to Table 2 and Table 3 and in the RESULT section of text.

26. Figure 1-2: Please provide exact p values, or indicate in the legend that these are reported elsewhere. Please also include the results from unadjusted analyses.

- Unadjusted data have been added to these figures. The p values can be found in the corresponding Tables 2 and 3.

27. Appendix 1 and Appendix 2: Please report exact p values, except for p<0.001, where applicable. Please also provide results from unadjusted analyses.

- This has been revised accordingly

Comments from the reviewers:

Reviewer #1: This study examines cross-sectional baseline data for associations between FEV1 with self-reported respiratory symptoms, perceived poor health, cognitive and physical performance.

Comments:

Can the authors please assess whether the comprehensive cohort with complete baseline data (22,822 participants) is representative of the wider nationally representative CLSA (51,338 participants)?

- A baseline characteristic table for the tracking and comprehensive cohort has been provided in Appendix 2 to allow comparison between the 2 cohorts, which showed similar baseline characteristics. This has been added to the RESULTS of the revised manuscript.

"Self-reported data from questionnaires included: age (45-54, 55-64, 65-74 & 75+ years); sex; smoking status (never [lifetime <100 cigarettes], former [last cigarette smoked >12 months], and current); education (below secondary, secondary and >secondary); known cardiovascular disease (CVD) (angina, congestive heart failure, myocardial infarction), chronic obstructive pulmonary disease (COPD), asthma and major chronic diseases (incorporated into the co-morbidity index 0, 1-2, >3). Height and weight were measured with standardized methods and equipment. Body mass index (BMI) was calculated as weight divided by height-squared and categorized into <25, 25-30, >30 kg/m2 . Self-reported physical activity was assessed by the Physical Activity Scale for the Elderly (PASE) questionnaire with higher weighted scores indicating higher activity levels in the previous 7 days."

Did the authors consider treating age and BMI as continuous variables (if available in this format from the questionnaires)?

- We did consider treating age and BMI as continuous variables. However, in case the relationship with lung function and outcome were not constant or monotonic throughout the range of these variables, we thought treating these variables as categories may accommodate any lack of constant relationship throughout the range of these variables.

Can the authors please acknowledge the potential bias of self-reporting, for both covariates and outcomes (e.g. perceived general health) in the study limitations?

- We have added the potential biases of self-reported outcomes in the DISCUSSION.

"The semantic fluency test assessed cognitive performance by asking participants to name as many animals within 60 seconds. Test scores were standardized for age, sex and education; and scores <45 considered impaired".

Did the authors consider treating cognitive assessment (i.e. semantic fluency test results) as a continuous variable?

Did the authors complete any sensitivity analyses on the cut-off of <45?

- A previous study has validated this threshold using the CLSA data. The reference has been added to the METHOD section.
  Findlay L, Bernier J, Tuokko H, Kirkland S, Gilmour H. Validation of cognitive functioning categories in the Canadian Community Health Survey--Healthy Aging. Health Rep. 2010 Dec;21(4):85-100. PMID: 21269015.

"Means and frequencies (%) were used to describe the sample. Hierarchical logistic regression was used to estimate the effects of low FEV1 (relative to FEV1>0sd as reference), adjusted for age, sex,
BMI, education, smoking status, self-reported asthma, COPD, CVD, co-morbidity index (excluding asthma, COPD and CVD). Centers were treated as random effect. The goodness-of-fit tests (likelihood ratio test, deviance, AIC, BIC criteria), multi-collinearity (tolerance and variance inflation factor) and visual inspection of residuals were performed to assess model stability and robustness. Trimmed inflation and analytical (rescaled) weights were applied; to reduce the effect of selection bias and maintain the national representativeness and generalizability of the data. Similar analyses after removing participants with spirometric obstruction (FEV1/FVC<LLN) were performed to ensure our findings were not confounded by diagnosed and undiagnosed COPD."

Did the authors examine the distribution of data, in order to confirm if mean or median may be more appropriate?

- We have examined the distribution of all the baseline variables and the means (sd) were most appropriate since FEV1, FVC and the continuous covariates were normally distributed on visual inspection of the histogram.

The authors should state in the methods section that they computed SD for continuous variables (as shown in Table 1).

- Thankyou for picking this up, we have added this information to the METHODS section.

Furthermore, the authors have completed suitable multilevel linear regression models to estimate the differences between each level of low FEV1 z-score relative to reference (FEV1%>0sd), but have not mentioned this in the methods section.

- This detail has been added to the METHODS.

"The prevalence and adjusted odds ratios (ORs) for these three outcomes showed a graded increase with lower FEV1 (Figure 1). The relationship was particularly strong (steep) between worsening FEV1 and increasing odds of moderate-severe respiratory symptoms, after accounting for differences in demographic and clinical factors. While the ORs were highest for the severely low FEV1 category; the absolute numbers of participants affected by these outcomes were far higher for the mild and moderately low FEV1 categories than for the severely low FEV1 group. "

Can the authors please quote accompanying relevant statistics in the main text, alongside each stated study finding?

- All relevant statistics have been added along side the stated findings in the RESULTS section.

Table 1:  Can the authors please complete statistical tests comparing baseline differences between groups, and present the results within this table ?

- Given the large sample size and multiple testing, we are concerned that these p values will most likely be statistically significant and may not be meaningful. However, we have adjusted for most of these baseline characteristics in Table 1 based on the likelihood of confounding by these factors.

Overall, the authors have appropriately applied multilevel modelling, suitably assessing model stability and exploring multi-collinearity.  Selection bias has been somewhat accounted for, and a thorough sensitivity analysis has been undertaken. The authors rightly acknowledge that the cross-sectional analysis limits the ability to infer causality, and that generalisability is restricted requiring findings to be examined in other populations.

Reviewer #2: Many epidemiologic studies have demonstrated that poor lung function, as indicated by a low forced expiratory volume in 1s (FEV1) and forced vital capacity (FVC), is associated with an increased risk of adverse outcomes, cardiovascular or pulmonary. The FEV1 indicator is included in frailty models. The reviewed article adds new information about its association with self reported symptoms, cognitive and physical performance in the general population. The researchers found a consistent and graded association between lower FEV1 with higher odds of self-reported poor health, moderate-severe respiratory symptoms and impaired cognitive performance, in a large representative sample of the general population. This results add that reduced lung function is a part of the generalized manifestations of functional and cognitive decline and potentially frailty. The results are important to the researches in geriatric, lung functions, cognitive disorders. There are the
implications for patient care and public policy.

In general the research is of very high methodological quality. The sample size is sufficient to prove the hypothesis. The data and analyses fully support the claims. The strength of the study is that large representative sample of the Canadian population aged 45 to 85 years living independently in the community was investigated. The large number of covariates collected allowed for careful adjustments to reduce any confounding effects.   The figures and tables are correct and of high technical quality. The manuscript is well organized and written clearly enough to be accessible to non-specialists.
I do not see any needs of major or minor improvement.

- We thankyou the reviewer for this positive review.

Reviewer #3: This is a cohort  of a population-based Canadian  about lung function  and the associated with higher mortality and adverse cardiopulmonary outcomes. The author analyse 22,822 adults (52% females, mean age 58.8 [sd 9.6]); the association between forced expiratory volume in 1 second (FEV1) with self-reported moderate to severe respiratory symptoms, perceived poor health, cognitive and physical performance were examined; using multilevel regression adjusted for potential confounders. The study is weel desging and the methdods and statitics sounds correct. The authors concluded that there is a  consistent and graded association between lower FEV1 with higher odds of self-reported poor health, moderate-severe respiratory symptoms and impaired cognitive performance, in a large representative sample of the general population. Similar gradient of associations were observed for physical performance on validated tests, which have important prognostication for future
functional impairment and poor health outcomes.
I have seen on the system three version of the article, I have read the R2 and I have any important consideration. There is no ethical and methodological issues. The introduction sounds good as well discussion and conclusioin that is machet with the objetctive of the article.

- We thankyou the reviewer for this positive review.

Reviewer #4: This study addresses an interesting association between low FEV1 and self reported "present health".  The findings are of interest but should be qualified.  Firstly the authors report an association and all implications of causality should be deleted (AGREED).  Secondly the participants were asked to "rate their present health" this is not the same as health status as measured by specific instruments.  It is not clear why one of the validated measures of health status was not used and tis should be discussed.  It would also be interesting to know if the findings also held true for the FVC measurements, as this would support the concept that the finding either reflects poor lung development or poor muscle strength rather than being related to airflow obstruction - the authors should present these data as well and discuss the results.

- The text and title have been revised to avoid implicating a causal relationship.
- Similar analyses on the FVC are presented in Appendix 8. Similar graded associations between different levels of FVC z scores with the same outcomes were observed. In fact for many of the outcomes, the effect sizes and strength of associations appear to be larger for FVC than FEV1. Furthermore, in our original analyses, we had excluded participants with obstructive impairment on spirometry, which further confirms that these associations are not limited to airflow obstruction. The larger effect sizes for the FVC suggest that mechanisms leading to reduced FVC such as poor lung development and muscle weakness may underly the association between reduced lung function with poor cognitive and physical performance and perceived general health. This has been added to the DISCUSSION.
