# Supplementary material for: Associations between lung function and physical and cognitive health in the Canadian Longitudinal Study on Aging (CLSA): A cross-sectional study from a multicenter national cohort
Source: PLoS Med. 2022 Feb 9;19(2):e1003909. doi: 10.1371/journal.pmed.1003909 (PMC8870596; doi:10.1371/journal.pmed.1003909)
Supplement: Attachment — Submitted filename: REPONSE to reviewers 23 Dec2021.docx [file pmed.1003909.s011.docx]

To the Editor

Re: PMEDICINE-D-21-01015R3

Manuscript title: "Associations between lung function with physical and cognitive health in the Canadian Longitudinal Study on Aging (CLSA), a multi-center population-based observational cohort study."

We thankyou for the opportunity to address the remaining editorial and production issues.

Please find below our response to each point raised with changes implemented accordingly.

Requests from Editors:

1. Financial disclosure: Please clarify the funding statement associated with your manuscript. You note both that “There was no funding support for the current analysis and work in this report.” but also list a number of funding sources as well as that “The funders had no role in study design, data collection and analysis, decision to publish, or preparation of this manuscript.”

Please address this discrepancy. This information should describe sources of funding that have supported the work included in this submission. The statement should include: Specific grant numbers, Initials of authors who received each award, and URLs to sponsors’ websites. Also state whether any sponsors or funders (other than the named authors) played any role in: Study design, Data collection and analysis, Decision to publish, Preparation of the manuscript. If they had no role in the research, include this sentence: “The funders had no role in study design, data collection and analysis, decision to publish, or preparation of the manuscript.” If the study was unfunded, include this sentence as the Financial Disclosure statement: “The author(s) received no specific funding for this work."

- The main study, CLSA was funded by the Canadian Institutes of Health Research (CIHR), with data that is accessible to the general scientific committee following a protocolized application and review access procedure. This work reported in this manuscript was made possible after applying and seeking approval for data access. This work was not funded and the authors volunteered their time to conduct and write up this manuscript. However, we do need to acknowledged CLSA for the data.
- Therefore, in the financial submission form we have indicated ‘The author(s) of this research received no specific funding for this work.’
- We have added an acknowledgement in the manuscript to indicate the following:

‘Funding for the main study, the Canadian Longitudinal Study on Aging (CLSA) is provided by the Government of Canada through the Canadian Institutes of Health Research (CIHR) under grant reference: CLSA 94473 and the Canada Foundation for Innovation. The CLSA is led by Drs. Parminder Raina, Christina Wolfson and Susan Kirkland. Dr Parminder Raina holds the Raymond and Margaret Labarge Chair in Optimal Aging and Knowledge Application for Optimal Aging, is the Director of the McMaster Institute for Research on Aging and the Labarge Centre for Mobility in Aging, and holds a Tier 1 Canada Research Chair in Geroscience. The funders had no role in study design, data collection and analysis, decision to publish, or preparation of the manuscript.

This research has been conducted using the CLSA dataset Baseline Comprehensive version 3.0, under Application Number 161013.

2. Data availability statement: Please update this section of the manuscript submission system with the details provided in the Data Access section of the main text (and please remove this section from the main text).

- The section of the manuscript submission system has been updated with the following details regarding data access: Data are available from the Canadian Longitudinal Study on Aging portal ([www.clsa-elcv.ca](http://www.clsa-elcv.ca)) for researchers who meet the criteria for access to de-identified CLSA data. The CLSA Data and Sample Access Committee is responsible for the review data access applications. Each application is reviewed for its relevance and feasibility. Applications for data access are available through an online process following this link: <https://www.clsa-elcv.ca/data-access/data-access-resources>.

3. Throughout: Please include line numbers running continuously throughout the text of the manuscript.

- Line numbers running continuously throughout the text of the manuscript has been added.

4. Abstract: Background We suggest revising to “...forced expiratory volume in 1 second (FEV1), an indicator of lung function, with broad markers of general health…”

- Above suggestion has been implemented.

5. Abstract: Methods and Findings: Please clarify what is meant by “Compared to the Global Lung Function Initiative predictive values” (could this be written as: “Based on Global Lung Function Initiative reference values”?).

- Above suggestion has been implemented.

6. Abstract: Methods and Findings: For the OR provided for perceived poor health and impaired cognitive performance, please indicate which of the three levels is being described for each (mild, moderate, severely low). Please also note that these are the adjusted OR, and please mention the factors adjusted for.

- The levels of FEV_1_ have been added for these outcomes. The covariates adjusted in the regression have also been added to this section of the Abstract.

7. Abstract: Methods and Findings, final sentence: Please revise to make the study limitations more clear: “A limitation is that the study is observational and causality cannot be inferred.” or similar.

- This phrase has been added to Methods and Findings.

8. Abstract: Conclusions: First sentence: Please remove the word “robust” from the sentence.
Author summary: Why was this study done? We suggest combining the first two bullet points.

- This has been revised as suggested.

9. Funding: Please remove the “Funding” section from the main text of the manuscript. Please ensure that all information is accurately entered into the manuscript submission system “Financial disclosure” section.

- This section has been removed from the manuscript and updated in the manuscript submission system.

10. Data Access: As mentioned above, please remove the “Data Access” section from the main text of the manuscript. Please ensure that all information is completely and accurately entered in the manuscript submission system in the “Data availability” section.

- This section has been removed from the manuscript and updated in the manuscript submission system in the ‘Data availability’ section.

11. Results: Please clarify this sentence “Compared to the overall cohort, the proportion of individuals with airflow obstruction whether defined by the GLI FEV1/FVC <LLN criterion (11% in overall vs. 58%), or FEV1/FVC<0.70 (5.4% vs. 45%) were higher in the severe FEV1 category.” We suggest noting as “11% in overall vs 58% in the severe FEV1 category” for example, if this is accurate.

- This sentence has been rephrased as follows: The overall prevalence of airflow obstruction defined by FEV_1_/FVC<0.70 was 11% (n=2,661) and by GLI FEV_1_/FVC <LLN was 5.4% (n=1,155). The prevalence of airflow obstruction increased with increasing level of FEV_1_ impairment, reaching as high as 58% (by the FEV_1_/FVC < 0.7 criterion) and 45% (by the GLI FEV_1_/FVC <LLN) among the severely low FEV_1_ category.

12. Results: Sensitivity analyses: Please note the location where the data are shown for the analyses where participants with airflow obstruction were removed (e.g. Table 2 and Table 3).

- Tables 2 & 3 have been added to this paragraph.

13. Discussion: First paragraph: There is a typo in the sentence: “Furthermore, they were These associations were highly consistent across different age groups…”

- This has been corrected.

14. Table 1: It would be helpful to provide a label at the top to indicate that the column headers indicate z score categories for GLI-FEV1 measures (this applies to all tables presented with z score column headers). Please define the abbreviation “FEV”

- The Title and a label have been rephrased/ added to all tables (including the Appendix) to clarify that the columns represent severity of impairment according to GLI-z scores.

15. Table 2 and Table 3: Please define the abbreviations for FFV, LLN, FVC in the legend.

- Definitions of these abbreviations have been added to the legend.

16. Figure 1: Please note in the legend the p values are reported in Table 2. Please provide a visual key within the figure indicating the definition of the open/closed circles and squares.

- The changes suggested have been implemented.

17. Figure 2: Please note in the legend the p values are reported in Table 3. Please provide a visual key within the figure indicating the definition of the open/closed circles and squares.

- The changes suggested have been implemented.

18. References: Please remove the italicized fonts from the reference list. Please check the formatting of each reference. Please note that there should be punctuation following the journal title. Please use the "Vancouver" style for reference formatting, and see our website for other reference guidelines <https://journals.plos.org/plosmedicine/s/submission-guidelines#loc-references>.

- The references in the manuscript and appendix have been formatted to comply with PLOS journal guidelines.

19. STROBE Checklist: Thank you for including the STROBE Checklist. Please revise to indicate the locations within the text using Section and Paragraph Numbers (e.g. Methods, paragraph 1). Please do not report page numbers or line numbers.

- The checklist has been revised accordingly.

20. Supporting Information files: List supporting information captions at the end of the manuscript file. Do not submit captions in a separate file. The file number and name are required in a caption, and we highly recommend including a one-line title as well. You may also include a legend in your caption, but it is not required. Authors may use almost any description as the item name for a supporting information file as long as it contains an “S” and number. For example, “S1 Appendix” and “S2 Appendix,” “S1 Table” and “S2 Table,” and so forth.

- A list of supporting information captions has been added at the end of the manuscript:

SUPPORTING FILES:

S1- Protocol of planned analysis submitted to Hamilton Integrated Research Ethics Board

S2- STROBE checklist

S3- Appendix 1. Baseline Characteristics of the comprehensive and tracking cohorts.

Appendix 2. Contraindications to performing spirometry:

Appendix 3. Analyses on self-reported respiratory symptoms, self-perceived poor health status, cognitive and physical performance for different grades of low FVC compared to reference (FVC>0sd) in the overall cohort and in participants without spirometry airflow obstruction (shown here as FEV1/FVC >=LLN).

Appendix 4. Unadjusted stratified analysis by gender, smoking history and baseline age for self-perceived poor health, respiratory symptoms and low cognitive scores by grades of low FEV1 relative to reference group (FEV1 >0sd).

Appendix 5. Adjusted stratified analyses by gender, smoking history and baseline age for self-perceived poor health, respiratory symptoms and low cognitive scores by grades of low FEV1 relative to reference group (FEV1 >0sd).

Appendix 6. Unadjusted stratified analyses by gender, smoking history and baseline age groups on physical performance according to grades of low FEV1 relative to reference group (FEV1 >0sd).

Appendix 7. Adjusted stratified analyses by gender, smoking history and baseline age groups on physical performance according to grades of lower FEV1 z scores relative to reference group (FEV1 >0sd).

21. Appendix 1: We suggest including the protocol as a separate file, and we suggest renaming to “S1_Protocol” or similar.

- Protocol will be submitted as a separate file labelled ‘S1- Protocol of planned analysis submitted to Hamilton Integrated Research Ethics Board’.

22. Appendix 2: In the legend, it would be helpful to briefly define what is meant by “comprehensive” and “tracking” cohort.

- This information has been added to Appendix 2.

23. Appendix 4: Please note that under “Cognitive Impairment” the categories for Smokers vs Non-smokers are repeated, and it seems the male/female stratification is missing.

- The labels were accidently switched and has been corrected.
